# Degree of suitability of information (DSI) for children with idiopathic scoliosis and their parents

**DOI:** 10.1186/1748-7161-9-S1-O72

**Published:** 2014-12-04

**Authors:** Elisabetta DAgata, Judith Sanchez-Raya, Joan Bago

**Affiliations:** 1 Fundacion Hospital Vall Hebron, Barcelona, Spain; 2 Hospital Vall Hebron, Barcelona, Spain

## Background

As parents and their children increasingly use Internet website to obtain information about Adolescent Idiopathic Scoliosis (AIS), a growing need to evaluate the content quality of websites is emerging. Bettany-Saltikov et al. and MacCulloch et al. have suggested some suitable requirements that websites on scoliosis should include. From these suggestions, we have created an instrument to quantify this suitability.

## Aim

To validate a specific instrument to assess the suitability of information for children with Idiopathic Scoliosis and their parents in English and Spanish websites.

## Design

Cross-sectional study. Validation of the psychometric properties of the instrument.

Methods: The DSI evaluates the degree the website oriented to parents and patients needs. The tool consists of 9 item with dichotomic answer (No=0 point, Yes=1); the range comes from 0 (worst quality) to 9 (best quality). It assesses the comprehension of the language used (item 1); different informartion for parents and children (2,3,4 items); the mentioning of different treatments (item 5) and quality of life themes (item 6); the availability of help service (item 7), graphic design (item 8) and web accessibility (item 9).To identify potential websites about scoliosis, the word “Scoliosis” in Spanish was used in five popular search engines (Google, Yahoo, Bing, Lycos, Ask). After excluding duplicates and videos, we obtained a list of 25 webs. Each web was evaluated separately by three observers. To evaluate the quality of medical information, DISCERN tool was used in addition to DSI.

Internal consistency was calculated. Intraclass correlation coefficient (ICC) was calculated to determine the intraobserver reliability of DSI. To assess convergent validity, correlation between DISCERN and DSI was calculated.

## Results

DSI mean was 4.3 (SD=1.8) and DISCERN mean was 38 (SD=4.3). For internal consistency, Cronbach’s α=0.6. ICC was 0.66. For convergent validity, r=0.7 (p<0.001).

## Conclusions

Although the internal consistency was not high and ICC value was moderate, DSI has good convergent validity. The questionnaire needs to be revised to maximize its psychometric properties (Content and Factorial Analysis). However it could be considered a new tool, short, specific to evaluate AIS websites and patient oriented.

